# Tularemia: The Resurgence of a Diagnostic Challenge and Clinical Dilemma in the United States

**DOI:** 10.7759/cureus.27363

**Published:** 2022-07-27

**Authors:** Michael Kelson, Justin Burnett, Sameh Girgis, Mohamed Bakr

**Affiliations:** 1 Psychiatry, Hackensack Meridian School of Medicine, Nutley, USA; 2 Anesthesiology, Drexel University College of Medicine, Philadelphia, USA; 3 Internal Medicine, Jersey Shore University Medical Center, Neptune, USA

**Keywords:** zoonotic infection, tick, ulcer, lymphadenopathy, epidemiology, infectious disease, rabbit fever, francisella tularensis, tularemia

## Abstract

Tularemia is a rare, life-threatening zoonotic infection with low, naturally occurring transmission rates in the United States. Classified as a Category A bioterrorism agent, this disease is highly infectious and has the potential to be fatal if left untreated. Making the diagnosis is difficult due to the non-specific symptomatology patients present with. Considering the increase in the prevalence of this diagnosis over the past two decades, this condition has the potential to become a public health crisis. This case report details a pediatric patient who was found to have fever, ulceration, and lymphadenopathy on hospital admission. After a prolonged and protracted course of illness, tularemia was diagnosed with laboratory testing. The purpose of this case report is to increase awareness of tularemia as a potential cause of non-specific flu-like symptoms, especially during the summer months. Moreover, our goal is to propose suggestions for healthcare professionals who encounter patients with clinical suspicion of tularemia.

## Introduction

Tularemia, commonly referred to as rabbit fever, is a zoonotic infection caused by the gram-negative bacterium *Francisella tularensis *[1]. This disease is usually transmitted to humans via tick bites, through direct contact with infected animals, and through inhalation of infectious aerosols [2]. This bacterium is highly contagious, needing as few as 10 microorganisms to cause a potentially fatal illness [1-3]. Thus, *F. tularensis* is one of six agents classified as a Category A bioterrorism disease, along with anthrax, botulism, plague, smallpox, and viral hemorrhagic fevers [4]. Considering the fact that this rare disease usually presents with non-specific viral symptomatology such as fever, malaise, chills, headache, and fatigue [5,6], it is of utmost importance to obtain a detailed exposure history for patients with suspected cases of tularemia so that appropriate diagnostic steps can be taken early in the course of illness. We present the case of a pediatric patient with a delayed diagnosis of ulceroglandular tularemia in the context of relapsing fever.

## Case presentation

A 13-year-old male with no significant past medical history presented to the hospital for four weeks of recurrent fevers and three weeks of worsening right upper extremity cellulitis with right fourth finger ulceration. As per the patient, his symptomatology began with a fever of 102° F, chills, nausea, vomiting, and what was initially thought to be a bug bite on the dorsum of his right fourth digit at the middle phalanx. Two days after the onset of these symptoms, the patient exhibited no improvement and thus sought evaluation by his primary care physician. The patient was prescribed oral cephalexin for seven days. After the course of treatment, the patient’s fever dissipated, however, he was now experiencing right arm swelling, erythema, and warmth to the medial aspect of his distal humerus. Approximately one week later, the patient’s fever recurs and he noticed that the right upper extremity erythema/swelling worsened which prompted him to visit the emergency department. In the ED, he was diagnosed with right fourth finger ulceration and right upper extremity lymphadenopathy. Bacterial (aerobic/anaerobic) wound cultures along with gram stain were obtained, which grew *Staphylococcus* *epidermidis*, and the patient was discharged with oral clindamycin. After five days of no clinical improvement, the patient went to his pediatrician’s office, who referred him to seek treatment at the hospital.

In the emergency department, the patient’s blood pressure was 119/67, heart rate 106/min, respiratory rate 18/min, oxygen saturation of 100% on room air, and temperature 101° F. He denied any nausea, vomiting, diarrhea, or cough at that time. On physical examination, the patient was in no apparent distress and was non-toxic appearing. As mentioned by the ED physician, a large ulcer was visible on the right fourth digit, measuring 6 x 6 cm, with swelling, firmness, induration, and surrounding granulation tissue. The swelling was also noted to the medial aspect of the right upper extremity with tenderness to palpation, warmth, and erythema. The patient was started on IV cefepime prior to admission to the pediatrics floor.

**Figure 1:**
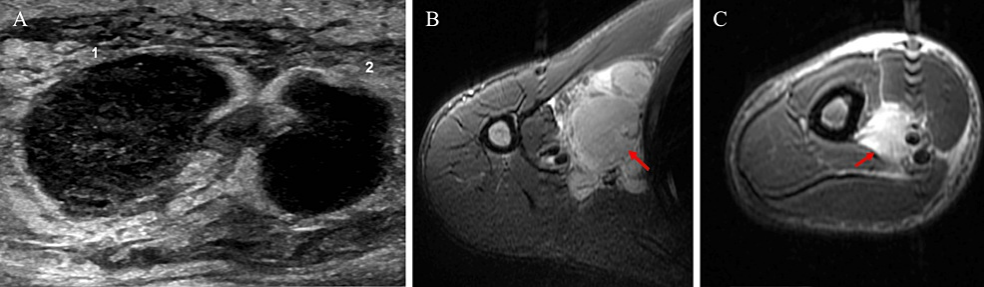
A: Ultrasound of the right distal humerus, bilobed structure measuring 3.1 x 1.2 cm, surrounding tissues are edematous; B: Magnetic resonance imaging (MRI) of the right humerus, axillary lymphadenopathy measuring 3.2 x 3.4 cm; C: MRI of the right humerus, enlarged lymph node measuring 3.0 x 2.7 cm

On the floor, IV vancomycin was initiated with resultant Red man syndrome which was stabilized with IV diphenhydramine. The patient’s fever at this time was controlled with oral acetaminophen. Labs came back abnormal with the following results: leukocyte count of 20.6 x 103/μL, platelet count of 582 x 103/μL, absolute neutrophil count of 15.4 x 103/μL, erythrocyte sedimentation rate of 36 mm/h, and C-reactive protein of 3.93 mg/dL. Serum electrolytes, liver function studies, blood urea nitrogen, creatinine, and lactate were within normal limits. Ultrasound imaging of the right humerus demonstrated phlegmonous lymph nodes with adjacent cellulitis, findings consistent with *Bartonella* *henselae* infection (Figure 1A). Magnetic resonance imaging (MRI) of the right humerus showed right axillary lymphadenopathy with the largest measuring 3.2 x 3.4 cm (Figure 1B-1C). Due to the aforementioned findings, orthopedic surgery and infectious disease were consulted. The patient’s fever recurred and he was noted to have fluctuance surrounding the right fourth finger ulceration and underwent incision and drainage for the abscess.

Due to recurrent episodes of fever, significant lymphadenopathy, and non-healing ulceration on the finger, the patient agreed to undergo surgery for lymph node biopsies. Post-operative findings were significant for right axillary and right epitrochlear necrotic lymph nodes and a right epitrochlear abscess. Further workup was negative for blood cultures, fungal cultures, viral cultures, deep wound cultures, acid-fast culture, *B. henselae* antibodies,* Blastomyces* antibodies, *Toxoplasma* IgM/IgG antibodies, *Histoplasma* urine antigen, and HSV polymerase chain reaction. However, *Francisella tularensis* antibodies came back positive (1:640). He was diagnosed with tularemia and started on IV gentamycin. After a five-week complicated course of tularemia infection, the patient’s fever resolved, the ulceration began to dry up, and he was discharged for home care with gentamycin via a peripherally inserted central catheter line.

## Discussion

Tularemia is a rare, life-threatening infection with low naturally occurring transmission rates in the United States [1,2]. Despite the low incidence rate of 2,108 cases in the United States from 2010 to 2019 (range: 124 - 314 cases/year) [7], tularemia remains on the list of nationally notifiable diseases due to being highly infectious with the potential to be utilized as a bioterrorism weapon [1-4]. One would think that a condition of this importance would be quick to diagnose, but due to its non-specific symptoms, the diagnosis can be delayed by even the most skilled clinicians. 

Tularemia can present in one of six different forms: ulceroglandular, glandular, oculoglandular, oropharyngeal, pneumonic, and typhoidal [2,5]. The incubation period from infectivity to symptom onset generally ranges between three days to three weeks, with most individuals experiencing flu-like symptoms and lymphadenopathy within the first week [8]. Its most common form, ulceroglandular tularemia, is usually caused by direct contact with rabbits or tick bites [1-3]. An ulcer forms at the site of inoculation and can persist for many months [2]. In contrast to this, the glandular form of tularemia presents with similar symptomatology but without ulcer formation [1,5]. The oculoglandular form is predominated by preauricular lymphadenopathy and conjunctivitis, while the oropharyngeal form presents with pharyngitis, stomatitis, and cervical lymphadenopathy [5]. The systemic forms of tularemia (pneumonic, typhoidal) are spread by the most virulent strain of this disease, with reported mortality rates of up to 60% [2,9]. Fortunately, pneumonic tularemia is rarely seen in clinical practice, however, the typhoidal form is present in 10-15% of cases [6,9]. 

As with the case of our patient, the clinical picture of tularemia demonstrates how a rare condition may not be at the forefront of a clinician’s differential diagnosis. Our patient presented with the classical non-specific signs of tularemia, which included relapsing fever, chills, nausea, vomiting, and an inoculation site on the right fourth digit at the middle phalanx [5,6]. His fever dissipated but returned along with lymphadenopathy of the upper extremity. Despite these symptoms, the patient’s diagnosis was delayed as is the case for many patients infected with *Francisella tularensis* [3]. Given that there are variable presentations of this illness which are based upon the route of infectivity and entry into the body, a detailed history is crucial in order to make the proper diagnosis of this condition [2,5,8]. We recommend that all healthcare professionals with any clinical suspicion of tularemia be proactive in asking patients about recent travel history, including outdoor activities such as hunting and camping, as well as groundwater or game meat ingestion. Likewise, patients should be questioned about recent contact with animals, given that this zoonotic pathogen finds itself a host in greater than 100 different animal species [1]. 

**Figure 2:**
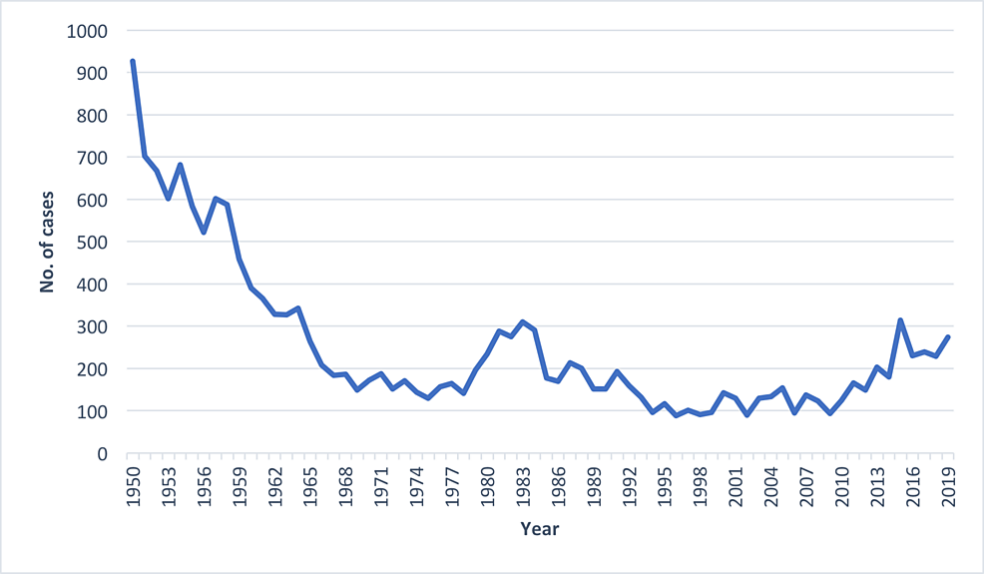
Incidence of confirmed tularemia cases in the United States, 1950-2019 Source: Centers for Disease Control and Prevention, National Center for Emerging and Zoonotic Infectious Diseases (NCEZID), Division of Vector-Borne Diseases (DVBD)

The importance of this case report is exemplified by the CDC statistics tracking data on the incidence of tularemia [7]. Since the 1950s, confirmed diagnostic cases have declined, with a resurgence of tularemia in the 1980s, and once again in the 2010s [7]. When viewing the data graphically from the past two decades (Figure 2), we can see that the number of cases per year continues to rise. From 2010 to 2019 there was a mean of 210.8 cases and a median of 216 cases per year. Taken alone, these statistics may not be alarming. However, a concerning picture is drawn when comparing those numbers to the years 2000-2009, which had nearly half as many cases, with a mean of 108.5 and a median of 129 [7].

The re-emergence of tularemia is a potential public health crisis in the making should delays in diagnoses continue [3]. Clinicians have difficulty with the diagnosis because of the vague non-specific symptoms and limited exposure they have to this condition in clinical practice [5,6]. We hope to bring awareness to tularemia’s rise in incidence over the past two decades as well as the complexities associated with recognizing this infectious disease. In order to combat its resurgence, we propose that physicians be made warier of the increased incidence during the summer months of May to September (79% of all cases from 2010-2019 in the United States) [7]. Likewise, all healthcare professionals should familiarize themselves with the most common presenting form of tularemia, the ulceroglandular subtype, which accounts for nearly 75% of all cases [1-3]. Additionally, a relapsing fever in the presence of a non-healing ulcer in an immunocompetent individual should prompt early serologic testing for this tick-borne illness. Antibodies may not be present early in the disease course, but repeated testing several weeks after the onset of initial symptoms will likely demonstrate seroconversion which can act as a confirmatory test [10]. Should seroconversion not occur, clinicians should suspect other etiologies which may present with similar symptomatology such as cat-scratch disease, plague, influenza, brucellosis, and infectious mononucleosis [6,9].

## Conclusions

Recognizing that tularemia can be a potential cause of non-specific systemic symptoms is vital for early identification and subsequent treatment of the underlying microbe. As this diagnosis is more prevalent during the summer months, clinicians should have a higher level of clinical suspicion during this time period. Moreover, given that global temperatures continue to rise with the progression of time, we should expect to see an increasing number of cases of tularemia in the future. Likewise, we anticipate that this zoonotic infection will occupy new geographic regions and find novel primary hosts. Thus, through this report, we hope to bring awareness to this re-emerging tick-borne infection. 

## References

[1] Petersen JM, Mead PS, Schriefer ME (2009). Francisella tularensis: an arthropod-borne pathogen. Vet Res.

[2] Yeni DK, Büyük F, Ashraf A, Shah MS (2021). Tularemia: a re-emerging tick-borne infectious disease. Folia Microbiol (Praha).

[3] Carvalho CL, Lopes de Carvalho I, Zé-Zé L, Núncio MS, Duarte EL (2014). Tularaemia: a challenging zoonosis. Comp Immunol Microbiol Infect Dis.

[4] Beale SL, Zolnikov TR, Firebaugh CM (2021). A scoping review on Category A agents as bioweapons. Prehosp Disaster Med.

[5] Snowden J, Simonsen KA (2022). Tularemia. https://www.ncbi.nlm.nih.gov/books/NBK430905/.

[6] (2022). WHO guidelines on tularemia. https://www.cdc.gov/tularemia/resources/whotularemiamanual.pdf.

[7] (2022). Tularemia Statistics. U.S. Department of Health & Human Services. https://www.cdc.gov/tularemia/statistics/index.html.

[8] Ellis J, Oyston PC, Green M, Titball RW (2002). Tularemia. Clin Microbiol Rev.

[9] Wawszczak M, Banaszczak B, Rastawicki W (2022). Tularaemia - a diagnostic challenge. Ann Agric Environ Med.

[10] Maurin M (2020). Francisella tularensis, tularemia and serological diagnosis. Front Cell Infect Microbiol.

